# Supplementary material for: Fabrication of Magnetic Al-Based Fe3O4@MIL-53 Metal Organic Framework for Capture of Multi-Pollutants Residue in Milk Followed by HPLC-UV
Source: Molecules. 2022 Mar 24;27(7):2088. doi: 10.3390/molecules27072088 (PMC9000854; doi:10.3390/molecules27072088)
Supplement: Supplementary file 1 [file molecules-27-02088-s001.zip › molecules-1641055-supplementary.pdf]

Supporting Information

Fabrication of magnetic Al-Based Fe_3O_4@MIL-53 metal organic framework for capture of multi-pollutants residue in milk followed by HPLC-UV

Authors: Xueli Liu[1,2], Yonghui Wang[2,#], Shuyue Ren[2], Shuang Li[2], Yu Wang[2], Dianpeng Han[2], Kang Qin[2], Yuan Peng[2], Tie Han[2], Zhixian Gao[2], Jianzhong Cui[1]*, Huanying Zhou[2]*

1 Department of chemistry, College of Science, Tianjin University, Tianjin, 300072, China.

2 Tianjin Key Laboratory of Risk Assessment and Control Technology for Environment and Food Safety, Tianjin Institute of Environmental and Operational Medicine, Tianjin 300050, China.

*** Corresponding Author:**

Email: cuijianzhong@tju.edu.cn (Prof. Jianzhong Cui).

zhouhytj@163.com (Prof. Huanying Zhou).

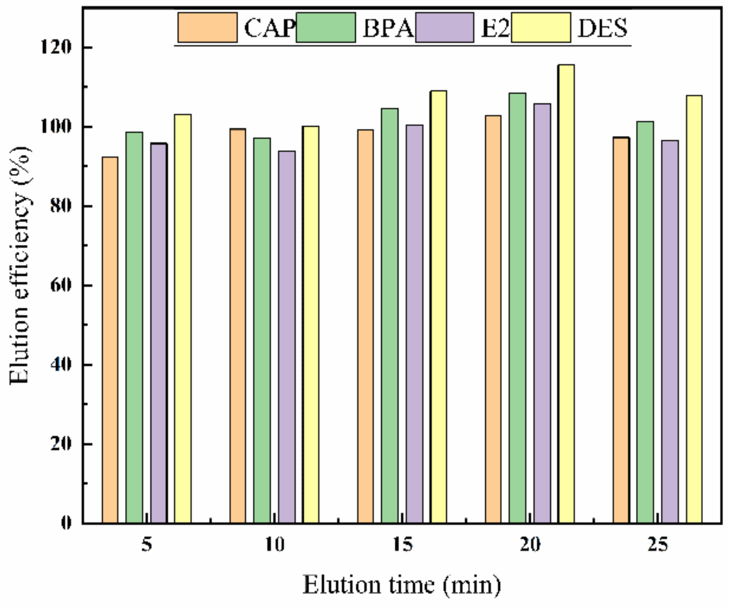

Fig. S1 Effect of elution time with 0.5mL acetonitrile for two times

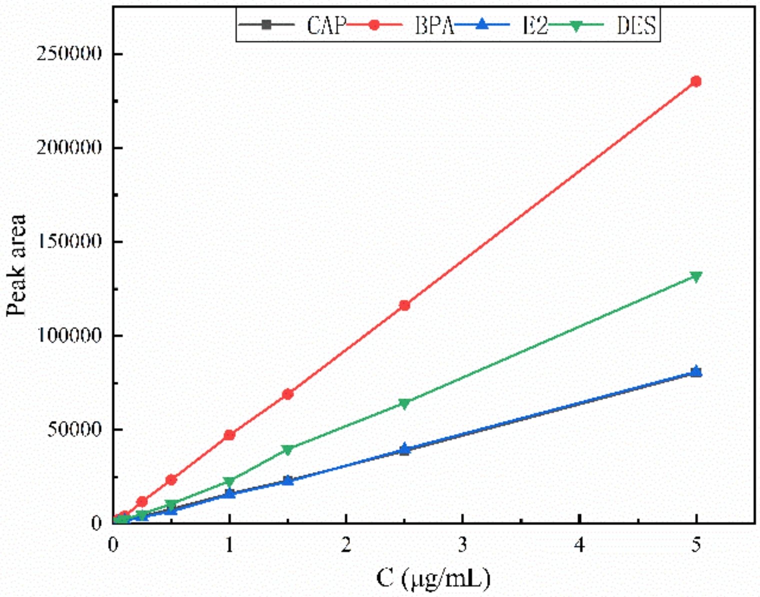

Fig. S2 The standard curve of four targets

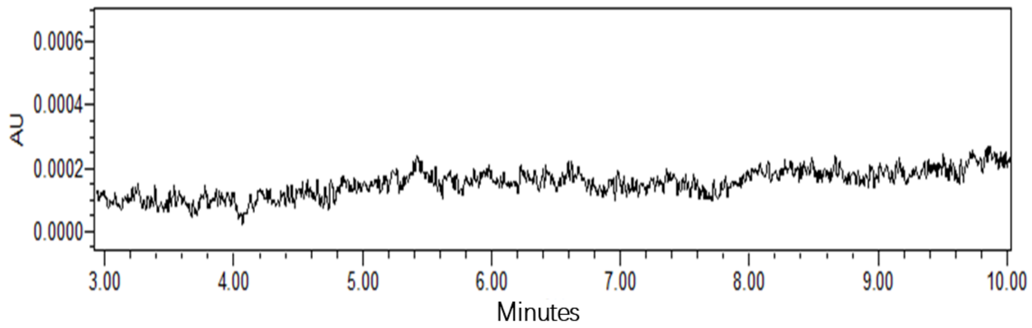

Fig. S3 HPLC chromatogram of empty milk

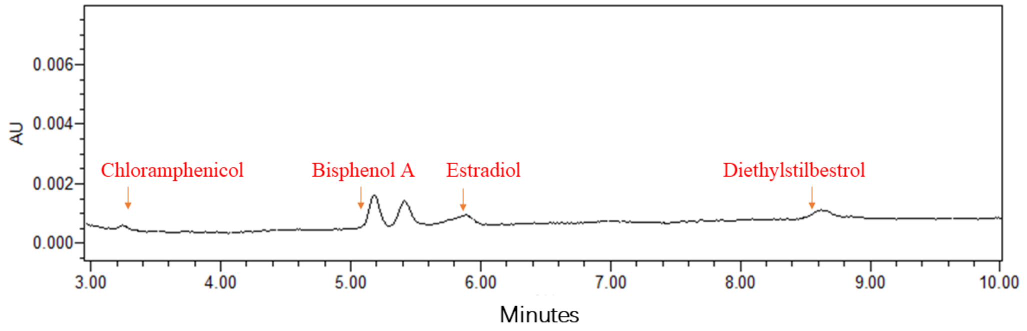

Fig. S4 HPLC chromatogram of positive milk sample at 0.15μg mL^{-1}

Table S1 The basic parameters of the target

Analytes	Formula	t_R(min)	Compound structure
CAP	$C_{11}H_{12}Cl_2N_2O_5$	3.2	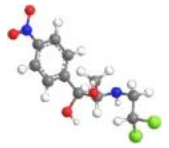
BPA	$C_{15}H_{16}O_2$	5.4	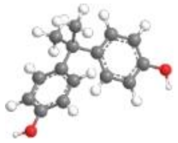
E2	$C_{18}H_{24}O_2$	5.9	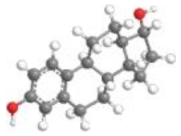
DES	$C_{18}H_{20}O_2$	8.7	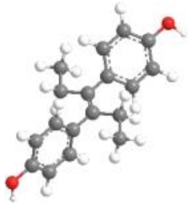

Table S2 Analytical performance of the proposed method

Analytes	Linear range ((μg mL^{-1})	R^2	LODs (μg mL^{-1})	LOQs (μg mL^{-1})	Repeatability (RSD %, n = 5)	
					Intraday	Interday
CAP	0.05-5	0.9995	0.108	0.113	0.23	1.14
BPA	0.05-5	0.9999	0.004	0.007	0.26	1.12
E2	0.05-5	0.9991	0.09	0.209	0.79	1.24
DES	0.05-5	0.9989	0.004	0.008	0.12	1.02
